# Nebulized Menthol Impairs Mucociliary Clearance via TRPM8 and MUC5AC/MUC5B in Primary Airway Epithelial Cells

**DOI:** 10.3390/ijms24021694

**Published:** 2023-01-15

**Authors:** Nathalie Baumlin, Neerupma Silswal, John S. Dennis, Asef J. Niloy, Michael D. Kim, Matthias Salathe

**Affiliations:** Department of Internal Medicine, Division of Pulmonary, Critical Care and Sleep Medicine, University of Kansas Medical Center, Kansas City, KS 66160, USA

**Keywords:** TRPM8, menthol, MUC5AC, MUC5B, airway epithelium

## Abstract

Flavorings enhance the palatability of e-cigarettes (e-cigs), with menthol remaining a popular choice among e-cig users. Menthol flavor remains one of the only flavors approved by the United States FDA for use in commercially available, pod-based e-cigs. However, the safety of inhaled menthol at the high concentrations used in e-cigs remains unclear. Here, we tested the effects of menthol on parameters of mucociliary clearance (MCC) in air–liquid interface (ALI) cultures of primary airway epithelial cells. ALI cultures treated with basolateral menthol (1 mM) showed a significant decrease in ciliary beat frequency (CBF) and airway surface liquid (ASL) volumes after 24 h. Menthol nebulized onto the surface of ALI cultures similarly reduced CBF and increased mucus concentrations, resulting in decreased rates of mucociliary transport. Nebulized menthol further increased the expression of mucin 5AC (MUC5AC) and mRNA expression of the inflammatory cytokines *IL1B* and *TNFA*. Menthol activated TRPM8, and the effects of menthol on MCC and inflammation could be blocked by a specific TRPM8 antagonist. These data provide further evidence that menthol at the concentrations used in e-cigs could cause harm to the airways.

## 1. Introduction

E-cigarettes (e-cigs) remain a popular alternative to combustible cigarettes, particularly among adolescents. In recent years, the United States Food and Drug Administration (FDA) has taken steps to regulate e-cigs and other electronic nicotine delivery systems (ENDS) to curb their use among young people. Flavors in e-cig liquids (e-liquids) appeal to youth, but flavoring chemicals have increasingly been shown to be harmful to the airway epithelium [1,2,3,4,5]. However, pod-based e-cigs remain widely available, with menthol and tobacco flavors being currently exempt from the FDA’s ban of flavored, pod-based e-cigs. Menthol flavor in particular was shown to be one of the most popular flavors among adolescent users of prefilled e-cig pods or cartridges such as JUUL [6]. Menthol is the dominant chemical in menthol-flavored e-cig pods, with concentrations exceeding 10 mg/mL (64 mM) in certain JUUL and disposable Puff Bar products [7]. Although menthol is purported to have beneficial effects as a decongestant and antitussive [8], whether inhalation of menthol at high concentrations in e-cigs is safe remains unclear.

Menthol is a cooling agent that primarily mediates its effects through activation of the transient receptor potential cation channel subfamily M (melastatin) member 8 (TRPM8) [9]. TRPM8 is a non-selective, calcium-permeable cation channel that is important for the detection of cooling sensations [9,10,11]. Although TRPM8 is predominantly found in peripheral sensory neurons, it is also expressed in the epithelium of the bladder, prostate, and the airways [12]. A truncated isoform of TRPM8 is expressed in normal human bronchial epithelial cells (HBEC) and was previously demonstrated to respond to cold temperatures as well as menthol at millimolar concentrations [13].

MUC5AC and MUC5B are the major mucins found in the airways [14,15]. Cold temperature and menthol were found to induce the expression of MUC5AC mRNA and protein in proliferating and submerged normal HBEC in a TRPM8-dependent manner [16]. Cold air was also shown to increase *MUC5AC* and decrease *MUC5B* mRNA expression in nasal cells of asthma patients [17]. It has been proposed that mucus concentration and the absolute concentrations of MUC5AC and MUC5B could be used as biomarkers of chronic bronchitis [18]. The importance of MUC5B, and its role in mucociliary clearance (MCC), was demonstrated in studies of *Muc5b* knockout mice, where MUC5B, but not MUC5AC, was found to be indispensable for normal airway clearance and infection control in the airways [19].

In addition to affecting mucins, activation of TRPM8 was also shown to increase the expression of inflammatory cytokines, including IL-6, IL-8, and TNF-α, in BEAS-2B and 16HBE human bronchial epithelial cell lines [13,20]. These studies point to an important role for menthol and TRPM8 in inducing inflammation and mucus production in the airways. However, whether menthol exerts other detrimental effects on MCC requires further investigation. In this study, we test the effects of relevant concentrations of basolateral and nebulized menthol on mucociliary function in primary HBEC fully differentiated at the air–liquid interface (ALI) by measuring ciliary beat frequency (CBF), mucus concentrations, and mucociliary transport (MCT). We show that menthol increases mucus concentrations and reduces both CBF and MCT and that this effect can be blocked by specific inhibitors to TRPM8. Furthermore, menthol increases MUC5AC expression, possibly contributing to mucociliary dysfunction.

## 2. Results

### 2.1. Menthol Increases TRPM8 mRNA Expression and Induces TRPM8-Mediated Ca^2+^ Responses

**Figure 1:**
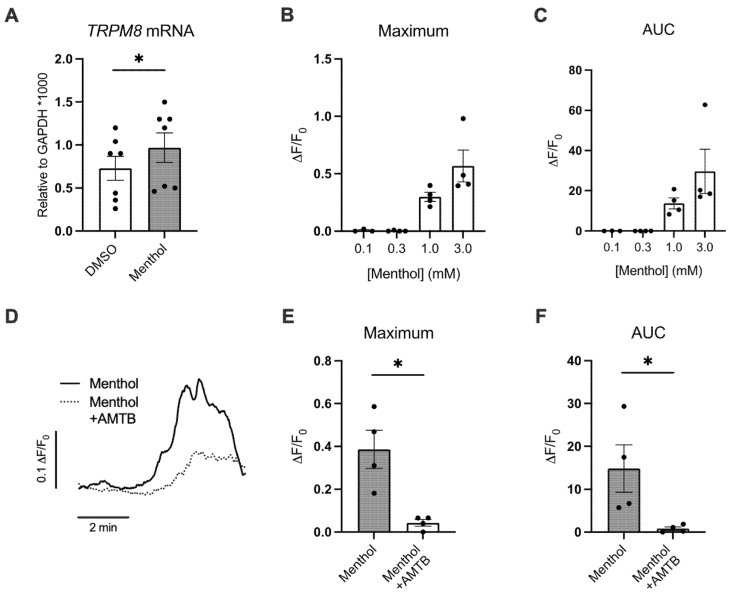
Menthol induces a Ca^2+^ response in primary HBEC via TRPM8. (**A**) Expression levels of *TRPM8* mRNA analyzed by ddPCR in HBEC treated with basolateral DMSO (0.1%) or menthol (1 mM) for 24 h. (**B,C**) Maximum (**B**) and area under the curve (AUC) (**C**) fluorescence changes caused by menthol (0.1, 0.3, 1.0, and 3.0 mM) in GCaMP6s-expressing HBEC. *n* ≥ 3, ≥2 lungs. (**D**) Representative tracing of GCaMP6s emission over time in HBEC exposed to menthol ± AMTB. (**E**,**F**) Maximum Ca^2+^ response (**E**) and AUC (**F**) fluorescence changes caused by menthol (1 mM) ± AMTB (30 µM). *n* = 4, 2 lungs. Statistics: Data shown as mean ± SEM. * *p* < 0.05, paired (**A**) or unpaired (**E**,**F**) *t*-test after assessing normality with Shapiro–Wilk.

*TRPM8* mRNA expression was found to be expressed in HBEC, as previously reported [13]. Basolateral treatment with menthol (1 mM) increased expression of *TRPM8* mRNA in primary HBEC compared to DMSO (0.1%) control (Figure 1A). *TRPM8* mRNA expression was detected using a TaqMan expression assay (Hs01066591_m1) that spanned the boundary between exons 22 and 23, consistent with a previous report detailing the expression of a truncated TRPM8 variant in airway epithelial cell lines [13]. We then investigated whether menthol activates TRPM8 receptors in HBEC by analyzing the dose-dependent response to menthol in HBEC expressing the fluorescent Ca^2+^ sensor GCaMP6s [21]. De-differentiated HBEC were transduced using a lentivirus construct containing the GCaMP6s sensor and selected with puromycin. The selected cells were allowed to fully differentiate for 4–6 weeks prior to the execution of the experiments. Basolateral perfusion of menthol led to a rapid increase in calcium at menthol concentrations ≥1 mM (Figure 1B,C), again confirming a response via the truncated TRPM8 variant. The calcium response to 1 mM menthol was blocked by the TRPM8 antagonist AMTB (30 µM; Figure 1D–F), suggesting TRPM8 can mediate menthol-induced changes in primary HBEC.

### 2.2. Menthol Reduces Ciliary Beating and ASL Volumes

**Figure 2:**
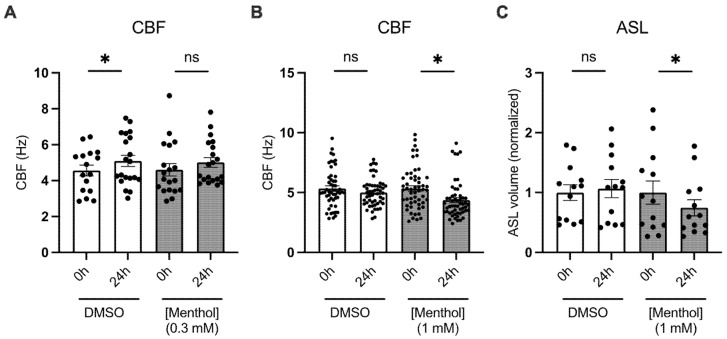
Effects of basolateral menthol on CBF and ASL volumes in primary HBEC. (**A**) CBF measured at 0 h and 24 h in HBEC treated with basolateral DMSO (0.1%) or menthol (0.3 mM). *n* = 16–20, 4 lungs. (**B**) CBF measured at 0 h and 24 h in HBEC treated with basolateral DMSO (0.1%) or menthol (1 mM). *n* = 52–55, 6 lungs. (**C**) ASL volume measured at 0 h and 24 h in HBEC treated with basolateral DMSO (0.1%) or menthol (1 mM). ASL volumes are normalized to baselines (0 h). *n* = 13, 8 lungs. Statistics: Data shown as mean ± SEM. * *p* < 0.05, two-way ANOVA followed by Holm–Sidak.

We next investigated whether menthol impacts parameters of mucociliary function in HBEC from non-smoking donors. Treatment of HBEC with basolateral menthol (1 mM) for 24 h caused a significant reduction in ciliary beat frequency (CBF; Figure 2). Menthol’s effects on CBF were only observed at menthol concentrations ≥1 mM (Figure 2A,B), consistent with its ability to activate TRPM8 in HBEC at these concentrations (Figure 1). Basolateral menthol (1 mM) for 24 h further caused a significant decrease in airway surface liquid (ASL) volumes compared to those of the controls (Figure 2C).

### 2.3. Nebulized Menthol Decreases CBF and Increases Inflammatory Markers

**Figure 3:**
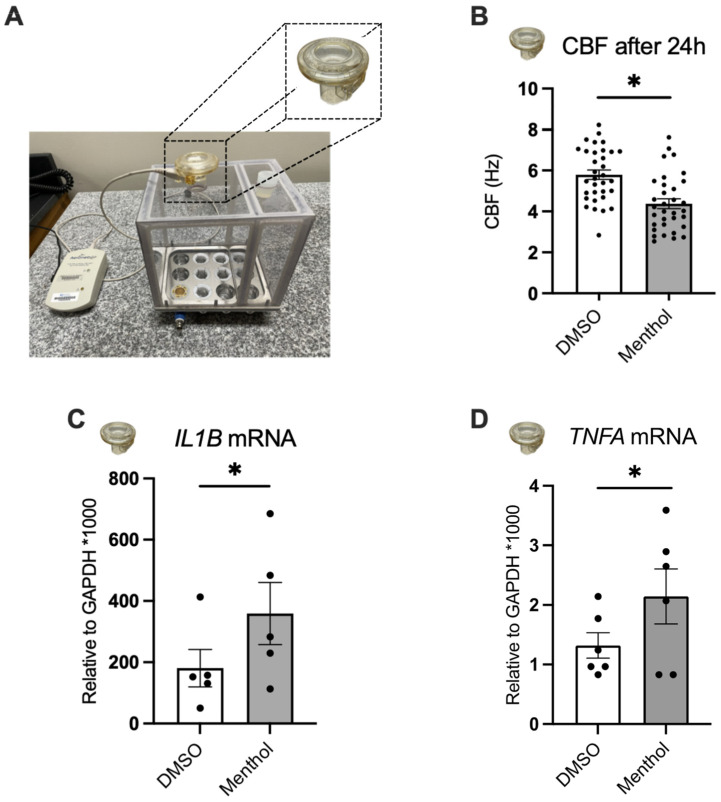
Effects of nebulized menthol on CBF and the expression of inflammatory markers in primary HBEC. All measurements were recorded 24 h after nebulization of DMSO (~0.1% final in the ASL, control) and menthol (~1 mM). (**A**) Cloud system with nebulizer. (**B**) CBF measured 24 h after menthol nebulization and DMSO control. *n* = 23, 5 lungs. (**C**) *IL1B* mRNA expression. *n* = 5, 3 lungs. (**D**) *TNFA* mRNA expression. *n* = 6, ≥3 lungs. Statistics: Data shown as mean ± SEM. * *p* < 0.05, mixed model analysis (**B**) or paired *t*–test (**C**,**D**) after assessing normality with Shapiro–Wilk.

To simulate e-cig exposure, we nebulized menthol onto the apical surface of HBEC using the Vitrocell Cloud (Figure 3A), as previously described [21]. The approximate concentration of menthol deposited onto the HBEC surface was estimated to be 1 mM, based on our previous studies [21]. Nebulized DMSO served as control (~0.1% final in the ASL). Measurements were conducted 24 h after exposure. Similar to basolateral treatment with menthol, we observed a significant decrease in CBF in menthol-exposed HBEC compared to DMSO controls (Figure 3B). Activation of TRPM8 induces inflammatory cytokines [20,22]. Nebulized menthol increased interleukin-1 beta (*IL1B*) and tumor necrosis factor alpha (*TNFA*) mRNA expressions compared to DMSO controls (Figure 3C,D).

### 2.4. Nebulized Menthol Alters Mucin Secretions

**Figure 4:**
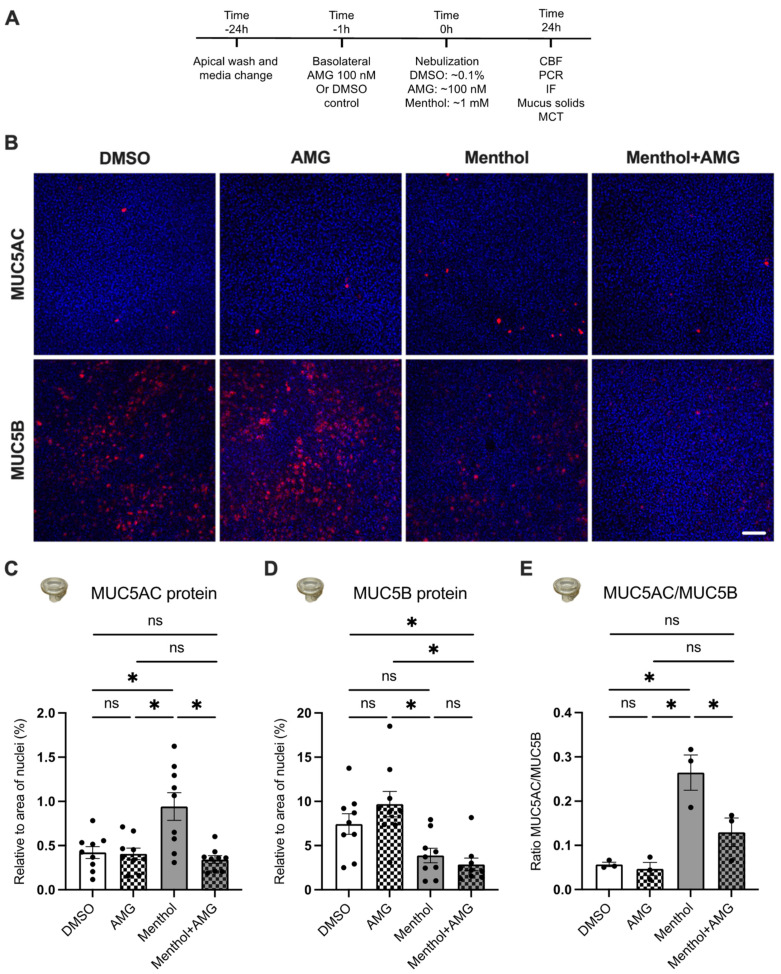
Effects of nebulized menthol and a TRPM8 antagonist on mucin expression in primary HBEC. (**A**) Schema of experimental design. All measurements were recorded 24 h after nebulization of DMSO (~0.1% final in the ASL, control) and menthol (~1 mM) with or without the TRPM8 antagonist AMG333 (~100 nM final in the ASL and 100 nM in the basolateral media). (**B**) Representative confocal images of MUC5AC and MUC5B (in red) as well as nuclei (Hoechst in blue). Scale bar represents 50 μm. (**C**,**D**) Quantification of expression of MUC5AC and MUC5B expressed as a ratio of surface area labeling of MUC5AC/Hoechst (**C**) and MUC5B/Hoechst (**D**), respectively. *n* = 9, 3 lungs. (**E**) Ratio of MUC5AC to MUC5B protein expression. *n* = 3 lungs. Statistics: Data shown as mean ± SEM. * *p* < 0.05, two–way ANOVA followed by Holm–Sidak (**B**,**C**) or one–way ANOVA followed by Holm–Sidak (**D**) after assessing normality with Shapiro–Wilk.

Menthol and inflammatory cytokines have been shown to increase airway mucins [17,23,24]. We, therefore, investigated the expression levels of the two major mucins found in the airways, namely MUC5AC and MUC5B [25]. Twenty-four hours after exposure to menthol or DMSO control, HBEC were stained with antibodies to MUC5AC and MUC5B (see Material and Methods) [26]. Airway epithelial cells that were positive for MUC5AC or MUC5B (both in red) and Hoechst (blue) were imaged using a Nikon C2+ confocal microscope (Figure 4) and quantified using ImageJ (Figure 4C–E). MUC5AC protein expression in HBEC ALI cultures was increased by menthol compared to controls (Figure 4C). Although menthol decreased MUC5B levels, the change was not statistically significant (Figure 4D). However, the overall MUC5AC/MUC5B ratio significantly increased in menthol-exposed HBEC versus controls (Figure 4E).

### 2.5. Blocking TRPM8 Prevents Menthol-Induced Increases in MUC5AC Expression, Reductions in CBF, and Increases in IL1B and TNFA mRNA Expressions

To determine whether menthol causes mucociliary dysfunction via TRPM8, we pre-treated HBEC with the TRPM8 antagonist AMG333 (100 nM in the basolateral media) 1 h before nebulizing DMSO, AMG, and menthol (Figure 4A). AMG333 was selected based on the nature of the experiments and its low IC50 [27]. Measurements were recorded 24 h after exposures. AMG333 prevented menthol-induced increases in MUC5AC levels but did not significantly impact MUC5B levels (Figure 4A–C). Importantly, the elevated MUC5AC/MUC5B ratio induced by menthol was rescued by AMG333 (Figure 4D).

**Figure 5:**
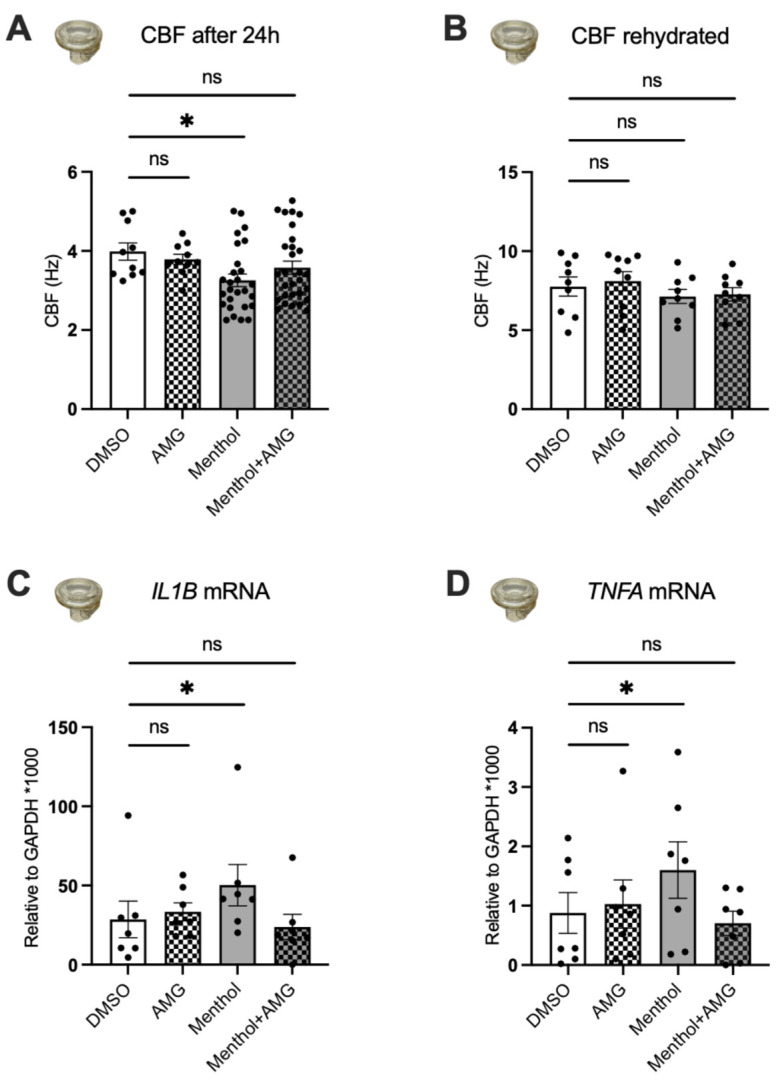
Effects of a TRPM8 antagonist on menthol-induced reductions in CBF and increases in *IL1B* and *TNFA* mRNA expressions. All assays were recorded 24 h after nebulization of DMSO (~0.1% final in the ASL, control) and menthol (~1 mM) with or without AMG333 (~100 nM final in the ASL and 100 nM in the basolateral media). (**A**) CBF measurements after nebulization of menthol and AMG333. *N* = 10–29, 2–6 lungs. (**B**) CBF measurements after rehydrating the surface of cultures with PBS. *N* = 9, 3 lungs. (**C**,**D**) Expression levels of *IL1B* (**C**) and *TNFA* (**D**) mRNAs. *N* = 7, 3 lungs. Statistics: Data shown as mean ± SEM. * *p* < 0.05 compared to DMSO control, two–way ANOVA followed by Dunnett (**A**,**B**) and one–way ANOVA followed by Dunnett (**C**,**D**).

Menthol significantly decreased CBF compared to DMSO controls (as shown above), and AMG333 prevented this reduction (Figure 5A). To show that menthol had no direct effect on cilia or their motor function, we rehydrated the epithelial surface with PBS. After rehydration, CBF was restored in the menthol-exposed HBEC, demonstrating that the reduction in CBF was likely caused by a decrease in periciliary fluid height (Figure 5B). Next, we measured expression of *IL1B* and *TNFA* mRNAs after exposure to aerosols of menthol in the presence or absence of AMG333. We found that menthol significantly increased the expression of these two cytokines (Figure 5C,D). Furthermore, treatment with AMG333 reversed the effects of menthol and restored *IL1B* and *TNFA* mRNA expressions to the levels of the DMSO controls. AMG333 treatment alone did not change the expression levels of *TNFA* or *IL1B* mRNA expressions.

### 2.6. Blocking TRPM8 Reverses Menthol-Induced Reductions in Additional Parameters of MCC

**Figure 6:**
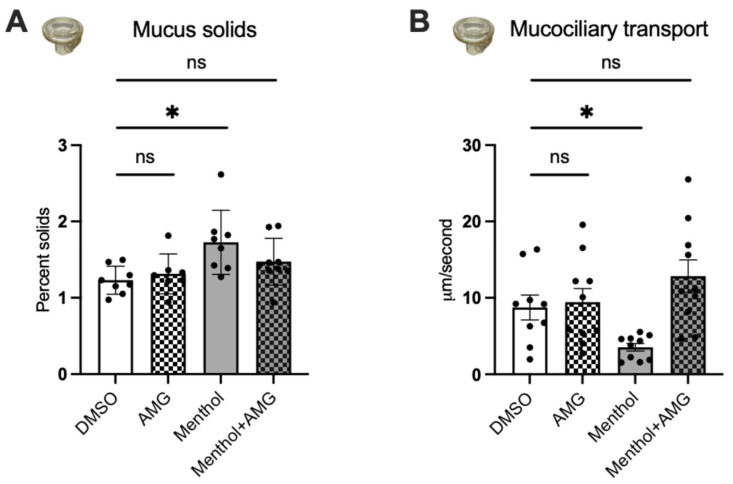
Effects of a TRPM8 antagonist on menthol-induced reductions in parameters of MCC. All measurements were recorded 24 h after nebulization of DMSO (~0.1% final in the ASL, control) and menthol (~1 mM) with or without AMG333 (~100 nM final in the ASL, and 100 nM in the basolateral media). (**A**) Percent mucus solids measured using a mesh. *n* ≥ 8 from 4 lungs. (**B**) Mucociliary transport measured with fluorescent beads. *n* ≥ 9 from 4 lungs. Statistics: Data shown as mean ± SEM. * *p* < 0.05 compared to DMSO control, two–way ANOVA followed by Dunnett (**A**) and one-way ANOVA followed by Dunnett (**B**).

Mucus concentrations (represented as percent solids) were measured with a mesh deposited on the HBEC cell surface and pulled off after incubation of 30 min at 37 °C. Menthol significantly increased mucus concentrations, which was prevented by AMG333 (Figure 6A), suggesting that inhibition of TRPM8 may reduce the viscosity of mucus. To confirm this, we measured mucociliary transport (MCT) by adding fluorescent beads 24 h after nebulization. Menthol significantly decreased rates of MCT compared to DMSO controls, but, again, this effect could be blocked by the TRPM8 antagonist AMG333 (Figure 6B).

## 3. Discussion

Increases in *IL6*, *IL8*, and *TNFA* mRNA expressions via TRPM8 were previously reported in BEAS-2B cell lines [13,20] and in 16HBE cell lines [22]. Cold air was also shown to increase the secretion of MUC5AC and decrease MUC5B mRNA expression in the nasal epithelium [13,20,28] and in 16HBE cell lines [22]. In this study, we confirmed some of these results in primary HBEC fully differentiated at the ALI but also revealed novel effects of menthol on MCC. We showed that basolateral treatment with menthol increases *TRPM8* mRNA expression and induces a TRPM8-mediated Ca^2+^ response. Basolateral menthol further causes significant decreases in ASL volumes and CBF. We observed similar results when HBEC were nebulized with menthol to simulate a more realistic exposure paradigm. Our data further showed menthol-induced TRPM8 activation, resulting in increased *IL1B* and *TNFA* mRNA expressions and in decreased CBF in our primary HBEC. While we did see an increase in MUC5AC protein expression, MUC5B was also decreased 24 h after nebulization of menthol (increasing the MUC5AC/MUC5B ratio). This was associated with an increased mucus concentration and decreased mucus transport, both features of mucociliary dysfunction.

Although short-term exposure of menthol was previously found to increase ciliary beating in human nasal epithelial cells [29], our data showed that longer menthol exposures can cause a significant decrease in CBF. Reduced CBF can be due to menthol directly affecting cilia functions or to a decrease in the periciliary layer (PCL) height, which can be caused by either ion channel dysfunction, increased mucus concentration, or both [30]. In order to narrow down which of these is most likely responsible for the decrease in CBF, we rehydrated the HBEC cultures and found fully recovered CBF, demonstrating that this effect was not due to direct ciliary damages, but rather due to a decrease in the PCL. Indeed, we found that mucus concentrations were significantly increased by menthol. These data, together with the increase in MUC5AC and decrease in MUC5B, show that the ratio of MUC5AC to MUC5B increased, likely further increasing mucus viscosity.

The composition of mucus (ratio of MUC5AC to MUC5B) is critically important for proper mucociliary function [25]. MUC5AC is produced by goblet cells on the surface of the epithelium, whereas MUC5B comes mainly from submucosal glands (some MUC5B is produced in surface goblet cells as well). MUC5B produced in larger quantities by submucosal glands is key to effective MCC [31]. MUC5AC can be tethered to the epithelial surface, worsening MCC [32]. Deletion of *muc5ac* in mice does not cause pulmonary problems in rodents, but deletion of *muc5b* was shown to have severe, negative consequences regarding infection control and MCC [19,32,33]. Furthermore, Costain et al. recently published a study which showed hereditary mucin deficiency with loss of MUC5B due to a novel splice variant in the MUC5B gene [34]. In the absence of MUC5B, the homozygous subject revealed extensive sinus disease and impaired MCC, while three heterozygous siblings were asymptomatic but revealed mild lung function impairments. In several airway diseases with mucociliary dysfunction, such as chronic bronchitis, the MUC5AC-to -UC5B ratio is increased [18]. These studies and our data showing menthol-induced increases in MUC5AC/MUC5B ratios led us to measure MCT in our cultures after menthol exposure. Rates of MCT were significantly reduced in the menthol-exposed HBEC and completely restored in the presence of the TRPM8 antagonist AMG333. Thus, the effects of menthol on mucin composition are likely to have an important influence on overall MCC.

E-liquids are largely composed of flavoring, nicotine, and the delivery vehicles propylene glycol (PG) and vegetable glycerin (VG). Nicotine alone can cause mucociliary dysfunction, impairing MCT and increasing mucus concentrations both in primary HBEC in vitro and sheep airways in vivo [21]. However, our recent study found that even e-cig vapors of only VG can increase mucus concentrations in the airway epithelium [35]. E-cig vapors comprising PG, VG, and tobacco flavor without nicotine were also shown to increase the expression of MUC5AC in cultures of human primary small airway epithelial cells [36]. The additive effects of these e-liquid constituents are likely to explain the increased expression of MUC5AC in bronchial epithelial cells and increased MUC5AC concentration, as well as the MUC5AC/MUC5B ratio in sputum samples of e-cig users [37,38].

Together, these data contribute to the accumulating body of evidence that menthol can cause harm in the airways, possibly via activation of TRPM8. There is increasing evidence of the importance of TRP channels in airway defense, but also of how dysregulation of TRP channels is linked to airway disease [39]. As nicotine can activate TRPA1 in the airway epithelium [21], and many popular flavorings are also potent agonists of TRP channels [40,41], it is important to understand how TRP channels contribute to the mucociliary dysfunction caused by e-cigs.

## 4. Materials and Methods

### 4.1. Chemicals

L-Menthol (cat. no. 125401000) was purchased from Acros Organics, Thermo Fisher Scientific, Waltham, MA, USA. N-(3-aminopropyl)-2-[(3-methylphenyl) methoxy]-N-(2-thienylmethyl) benzamide hydrochloride (AMTB; cat. no. 3989) and (S)-6-(((3-Fluoro-4-(trifluoromethoxy)phenyl)(3-fluoropyridin-2-yl)methyl)carbamoyl)nicotinic acid (AMG333; cat. no. 6874) were purchased from Tocris, Bio-Techne Corporation, Minneapolis, MN, USA.

### 4.2. Air–Liquid Interface (ALI) Cultures

All primary HBEC were harvested from non-smoking donors whose lungs were rejected for transplant and did not suffer from any known airway disease. Passage 0 (P0) cells were expanded in PneumaCult^TM^-Ex Plus Medium (STEMCELL Technologies, Cambridge, MA, USA) supplemented with gentamicin (Thermo Fisher Scientific, Waltham, MA, USA) and amphotericin B (Thermo Fisher Scientific). Passage 1 (P1) cells were expanded in bronchial epithelial cell growth medium (BEGM) before being seeded on Transwell cell culture inserts (cat# 3450, 3460, Corning, Glendale, AZ, USA) or Thincert (cat# 665640, Greiner, Monroe, NC, USA) at a density of 2 × 10^5^ cells in ALI media, as previously described [21]. HBEC were kept submerged in ALI media until reaching confluency, when the air–liquid interface was initiated (5–7 days). Media were replaced every other day, and the apical surface of the cultures was washed with Dulbecco’s phosphate-buffered saline (DPBS; Corning). The cells were allowed to differentiate for 4–6 weeks before all displayed experiments were carried out.

### 4.3. Calcium Imaging

Lentivirus was prepared by co-transfecting HEK293T cells with packaging DNA plasmids and a pEF1-puromycin-GCaMP6s construct [42]. De-differentiated basal cells were transduced with virus in ALI media containing polybrene, as described [42]. Cells were then allowed to fully differentiate at the ALI under puromycin selection (1 μg/mL, cat. no. A11138-03, Thermo Fisher Scientific). GCaMP6s-infected HBEC were mounted in a perfusion chamber placed on the stage of an upright Nikon Eclipse E600fn microscope (Nikon Instruments, Tokyo, Japan) and visualized with a 60x water immersion objective. Cells were perfused with menthol in HEPES-buffered HBSS pH 7.4 at 250 µL/min using a syringe pump (Harvard Apparatus, Holliston, MA, USA). Menthol was prepared by dissolving the crystals in DMSO to create a stock solution. When the solutions used in the perfusion were prepared, the menthol stock solution was diluted in the appropriate HEPES-buffered HBSS pH 7.4. Images were acquired every 3 s with a Prime BSI Express camera (Teledyne Photometrics, Tucson, AZ, USA) after GCaMP6s excitation (495 nm) with a Lambda DG-4 high speed wavelength switcher (Sutter Instrument, Novato, CA, USA) controlled by MetaFluor^®^ software (Molecular Devices, San Jose, CA, USA).

### 4.4. Airway Surface Liquid (ASL) Volumes

Meniscus scanning of high-resolution images of ALI cultures was used to estimate ASL volumes, as previously described [43,44]. Briefly, the HBEC culture plate was placed on an Epson scanner at room temperature, lid off, and a humidity of at least 50–60% to avoid dehydration during the recording. ASL volume estimations were recorded at time 0 h (prior to nebulization) and 24 h after nebulization. Data were analyzed using ImageJ and Dr. Myerburg’s software, as published before.

### 4.5. Ciliary Beat Frequency (CBF)

CBF of primary HBEC was recorded using a high-speed Basler acA645 camera (Basler, Ahrensburg, Germany) mounted on a Zeiss Axiovert 200M (Carl Zeiss, Jena, Germany) running SAVA software, as previously described [26,45]. Briefly, the HBEC culture plate was mounted on the stage of the microscope, and five videos were recorded for each culture well at room temperature at time 0 h (pre) and 24 h (post nebulization).

### 4.6. Nebulization of Menthol and AMG333 via the Cloud System and Media Treatment

HBEC cultures were aerosolized with defined solutions supplemented with PBS using the Vitrocell Cloud system (Vitrocell, Waldkirch, Germany), as previously described [21,46]. Menthol crystals were dissolved in DMSO to make a stock solution of 1 M. An aliquot of 200 µL of a 100 mM menthol solution via dilution in PBS was then nebulized onto the surface of the cultures with an estimated deposition of 1 mM menthol. For AMG333 experiments, a stock solution of 100 mM was prepared in DMSO, and 200 µL of a 10 µM AMG333 solution via dilution in PBS was nebulized onto the surface of the cultures with an estimated deposition of 100 nM AMG333. As controls, and to balance fluid addition (with appropriate DMSO concentrations), an additional 200 µL of appropriate concentrations of DMSO in PBS was nebulized onto the surface of the cultures to ensure that all wells received the same amount of nebulized fluid and the same DMSO concentration.

Final DMSO concentrations on the surface of the culture or in culture media did not exceed 0.1% (so had no measurable effect on these cells). All assays were performed 24 h after nebulization. In addition, 100 nM of AMG333 was added into the media 24 h before nebulization.

### 4.7. Immunofluorescence

Immunofluorescence staining of HBEC after nebulization was performed as described [26]. Membranes were fixed with a solution of 50%/50% methanol/acetone for 2 min at −20 °C followed by three washes with PBS containing 0.05% Tween 20. A 3% BSA solution was used to block for 1 h before incubation with anti-MUC5AC (cat# MA512178; Thermo Fisher Scientific) and anti-MUC5B (cat# PA82342; Thermo Fisher Scientific) antibodies at 0.2 μg/mL in PBS containing 0.05% Tween 20 overnight at 4 °C. The next day, the membranes were washed three times with PBS containing 0.05% Tween 20 before incubation with secondary antibody donkey anti-mouse Alexa Fluor 555™ (cat# A31570; Thermo Fisher Scientific) or donkey anti-rabbit Alexa Fluor 555™ (cat# A31572; Thermo Fisher Scientific) at 0.4 μg/mL in PBS containing 0.05% Tween 20 for 1 h at room temperature. After the secondary antibody, membranes were washed again three times in PBS containing 0.05% Tween 20 before adding Hoechst (cat# H3569; Thermo Fisher Scientific) at 2 μg/mL for 10 min at room temperature in PBS containing 0.05% Tween 20. Membranes were mounted onto slides using Fluoro-Gel mounting media (cat# 17985-11; Electron Microscopy Sciences, Hatfield, PA, USA). All slides were imaged with a Nikon C2+ confocal microscope (Nikon Instruments, Japan), and fluorescent intensity measurements were performed with ImageJ (Bethesda, MD, USA), as previously described [26].

### 4.8. Mucus Solids Measurements

The percentage of solids of mucin-containing fluid was measured according to published methods of mucus wet and dry weights using a UMX2 ultra-microbalance with an accuracy of 100 ng (Mettler Toledo, Columbus, OH, USA) [26]. Briefly, a laser-cut mesh was first weighed in an aluminum boat and recorded as “pan weight” using Igor Pro 8 (WaveMetrics, Inc., Portland, OR, USA). Second, the mesh was deposited onto the surface of the cultures and incubated for 30 min at 37 °C in a CO_2_ incubator, then lifted off, put back into the aluminum boat, and immediately recorded as “wet weight”, while the clock was started at the moment the mesh came off in order to calculate back the exact wet weight. Third, the aluminum boat was incubated overnight in an oven at 65 °C before being measured again and recorded as “dry weight”. Data were analyzed using Igor Pro 8 and represented as percent solids.

### 4.9. Mucociliary Transport (MCT) Measurements

HBEC donors were differentiated on Transwell inserts in ALI media for 6–8 weeks to develop continuous MCT, as described previously [21]. Briefly, fully differentiated HBEC were nebulized with menthol, DMSO, and/or AMG333. After 24 h, MCT rate was recorded by apically applying 10 µL of fluorescent microbeads (1:10,000 dilution in 1× PBS) and incubating for 10 min at 37 °C in a CO_2_ incubator, allowing the beads to equilibrate. FluoSpheres™ Carboxylate-Modified Microspheres (cat# F8823) were purchased from Thermo Fisher Scientific. The velocity for each treatment was recorded every 3 s for 30 s at an emission of 515 nm. The Manual Tracking ImageJ plugin was used to analyze and quantify the results.

### 4.10. Quantitative PCR (qPCR) and Droplet Digital PCR (ddPCR)

HBEC were lysed 24 h after exposure, and total RNA was isolated using the E.Z.N.A.^®^ Total RNA kit (Omega Bio-tek, Norcross, GA, USA). qPCR or ddPCR was performed using TaqMan Gene Expression Assays for *IL1B* (Hs01555410_m1), *TNFA* (Hs00174128_m1), and *TRPM8* (Hs01066591_m1) and normalized to reference gene *GAPDH (*4326317E). ddPCR was performed as previously described [47].

### 4.11. Statistics

All data are presented as mean ± SEM and were considered statistically significant when *p* < 0.05. The Shapiro–Wilk method was used to test for normality. Paired or unpaired *t*-test or mixed model analysis was used to compare two groups. One-way ANOVA followed by Holm–Sidak or Dunnett or two-way ANOVA followed by Holm–Sidak or Dunnett was used to compare multiple groups.

## Data Availability

The data presented here are available upon request from the corresponding author.
